# A prospective study of bloodstream infections among febrile adolescents and adults attending Yangon General Hospital, Yangon, Myanmar

**DOI:** 10.1371/journal.pntd.0008268

**Published:** 2020-04-30

**Authors:** Tin Ohn Myat, Khine Mar Oo, Hla Kye Mone, Wah Win Htike, Ambarish Biswas, Rachel F. Hannaway, David R. Murdoch, James E. Ussher, John A. Crump

**Affiliations:** 1 Department of Microbiology, University of Medicine 1, Yangon, Myanmar; 2 Centre for International Health, University of Otago, Dunedin, New Zealand; 3 Bacteriology Section, National Health Laboratory, Yangon, Myanmar; 4 Department of Microbiology and Immunology, University of Otago, Dunedin, New Zealand; 5 Department of Pathology and Biomedical Science, University of Otago, Christchurch, New Zealand; 6 Southern Community Laboratories, Dunedin, New Zealand; Institute for Disease Modeling, UNITED STATES

## Abstract

Data on causes of community-onset bloodstream infection in Myanmar are scarce. We aimed to identify etiological agents of bloodstream infections and patterns of antimicrobial resistance among febrile adolescents and adults attending Yangon General Hospital (YGH), Yangon, Myanmar. We recruited patients ≥12 years old with fever ≥38°C who attended YGH from 5 October 2015 through 4 October 2016. A standardized clinical history and physical examination was performed. Provisional diagnoses and vital status at discharge was recorded. Blood was collected for culture, bloodstream isolates were identified, and antimicrobial susceptibility testing was performed. Using whole-genome sequencing, we identified antimicrobial resistance mechanisms of *Enterobacteriaceae* and sequence types of *Enterobacteriaceae* and *Streptococcus agalactiae*. Among 947 participants, 90 (9.5%) had bloodstream infections (BSI) of which 82 (91.1%) were of community-onset. Of 91 pathogens isolated from 90 positive blood cultures, we identified 43 (47.3%) *Salmonella enterica* including 33 (76.7%) serovar Typhi and 10 (23.3%) serovar Paratyphi A; 20 (22.0%) *Escherichia coli*; 7 (7.7%) *Klebsiella pneumoniae*; 6 (6.6%), *Staphylococcus aureus*; 4 (4.4%) yeasts; and 1 (1.1%) each of *Burkholderia pseudomallei* and *Streptococcus agalactiae*. Of 70 *Enterobacteriaceae*, 62 (88.6%) were fluoroquinolone-resistant. Among 27 *E*. *coli* and *K*. *pneumoniae*, 18 (66.6%) were extended-spectrum beta-lactamase (ESBL)-producers, and 1 (3.7%) each were AmpC beta-lactamase- and carbapenemase-producers. Fluoroquinolone resistance was associated predominantly with mutations in the quinolone resistance-determining region. *bla*_CTX-M-15_ expression was common among ESBL-producers. Methicillin-resistant *S*. *aureus* was not detected. Fluoroquinolone-resistant, but not multiple drug-resistant, typhoidal *S*. *enterica* was the leading cause of community-onset BSI at a tertiary hospital in Yangon, Myanmar. Fluoroquinolone and extended-spectrum cephalosporin resistance was common among other *Enterobactericeae*. Our findings inform empiric management of severe febrile illness in Yangon and indicate that measures to prevent and control enteric fever are warranted. We suggest ongoing monitoring and efforts to mitigate antimicrobial resistance among community-onset pathogens.

## Author summary

Bloodstream infection (BSI) is common among persons seeking healthcare for severe febrile illness in low-and middle-income countries. Data on community-onset BSI are few for some countries in Asia, including Myanmar. Such data are needed to inform empiric antimicrobial treatment of patients and to monitor and control antimicrobial resistance. We performed a one year, prospective study collecting information and blood cultures from patients presenting with fever at a tertiary referral hospital in Yangon, Myanmar. We found that almost 10% of participants had a bloodstream infection, and that *Salmonella enterica* serovars Typhi and Paratyphi A were the most common pathogens. Typhoidal *Salmonella* were universally resistant to ciprofloxacin. More than half of *Escherichia coli* and *Klebsiella pneumoniae* were resistant to extended-spectrum cephalosporins and resistance to carbapenems was also identified in some isolates. We show that typhoid and paratyphoid fever are common, and fluoroquinolone resistance is widespread. Extended-spectrum cephalosporin resistance is common in *E*. *coli* and *K*. *pneumoniae* and carbapenem resistance is present. Our findings inform empiric antimicrobial management of severe febrile illness, underscore the value of routine use of blood cultures, indicate that measures to prevent and control enteric fever are warranted, and suggest a need to monitor and mitigate antimicrobial resistance among community-acquired pathogens.

## Introduction

Fever is a common reason for seeking healthcare in South-East Asia [1, 2]. Bloodstream infection (BSI) is an important cause of severe febrile illness [3] and requires urgent and appropriate antimicrobial therapy to avert death [4]. The causes of bacteremia and patterns of antimicrobial resistance among bloodstream isolates may vary considerably by location and change over time. Data are needed to inform empiric antimicrobial regimens and to identify emerging pathogens and antimicrobial resistance problems. A 2012 systematic review of community-acquired bloodstream infections in South and South-East Asia identified just 17 studies [5], and only one from Myanmar [6], indicating major data gaps in the region.

Lack of clinical microbiology services is an important barrier to understanding BSIs in low-resource areas [7]. Where blood cultures can be performed, they may be subject to quality concerns or may not be obtained systematically from patients who could potentially benefit. Furthermore, laboratory records frequently lack sufficient detail to make the epidemiologically important distinction between hospital-acquired and community-onset infections. Antimicrobial resistance is a growing problem globally and especially in South and South-East Asia among hospital-acquired infections [8]. Moreover, community-acquired pathogens with concerning patterns of antimicrobial resistance, including extended-spectrum cephalosporin-resistant *S*. *enterica* Typhi [9] and other *Enterobacteriaceae* [10–12], are increasingly identified.

In order to improve knowledge on the epidemiology of community-onset BSI in Myanmar, we sought to identify the bacterial and fungal etiology, and antimicrobial susceptibility of bloodstream isolates from adolescent and adult patients with febrile illness attending the Yangon General Hospital (YGH), Yangon, Myanmar. We also determined genetic mechanisms of antimicrobial resistance among *Enterobacteriaceae* bloodstream isolates.

## Methods

### Ethics statement

The study protocol was reviewed and approved by Ethics Review Committees of University of Medicine 1, and the Department of Medical Research, Yangon, Myanmar, and the Human Ethics Committee of the University of Otago (reference number: H15/045). We sought and obtained written informed consent from guardians or caregivers for patients aged between 12 to 18 years of age, those who were illiterate, or were unconscious at presentation. For all others, written consent was sought and obtained from the patient.

### Setting

**Fig 1:**
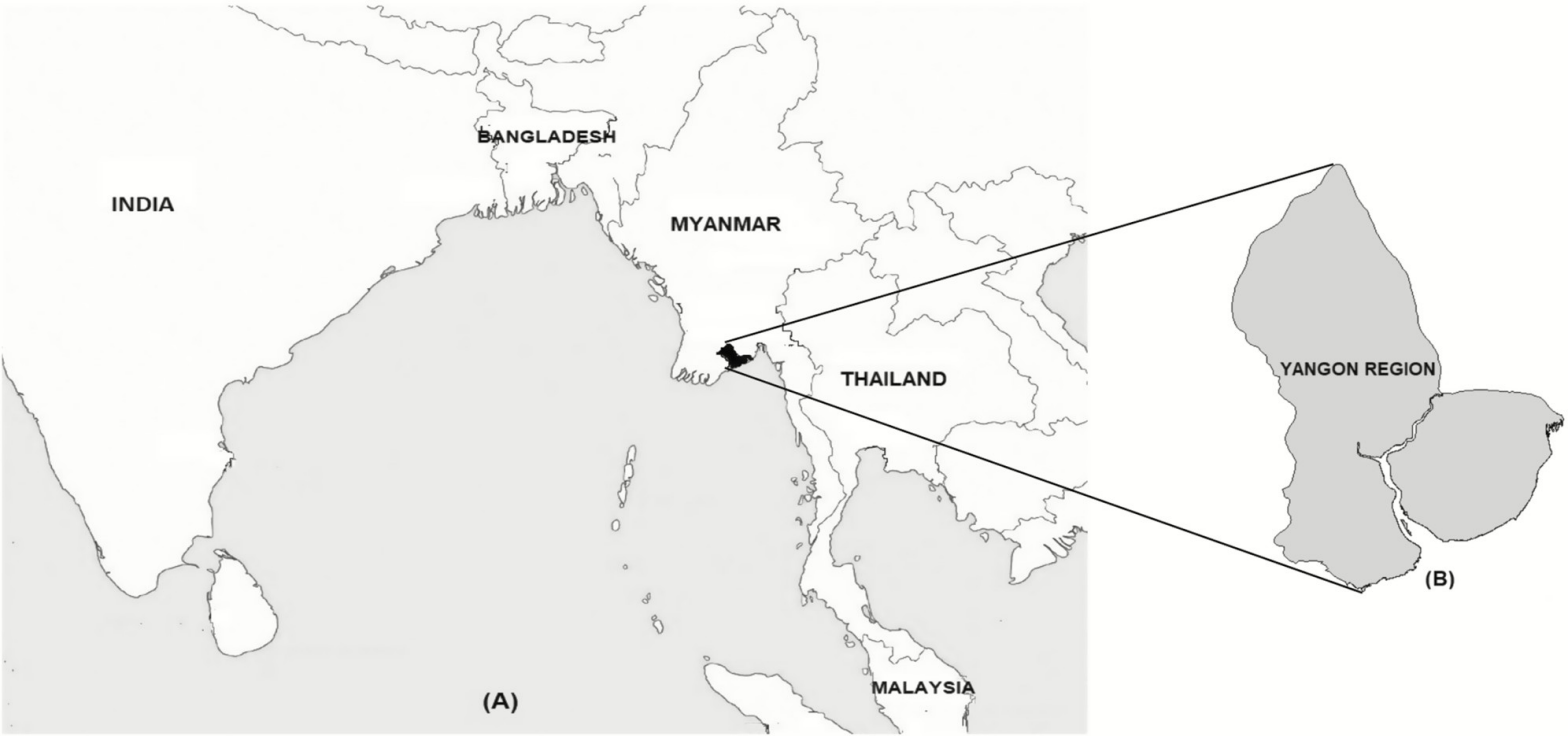
Map of South and South-East Asia showing Myanmar (panel A) and Yangon Region (panel B). Reprinted from Oo WT et al [14].

Yangon, the largest city and former capital of Myanmar, is situated in the Yangon Region (Fig 1) with a population of 5.16 million [13]. YGH is a 2,000-bed hospital and is the largest civilian tertiary referral hospital in Myanmar receiving patients directly from the community as well as by referral from other hospitals nationwide. YGH provides free medical and surgical care services to outpatients and inpatients aged ≥12 years. Following triage in the Department of Emergency Medicine, febrile patients without surgical conditions are referred to the Medical Observation (MO) Unit for pre-admission care or referral for outpatient management.

### Participants

We prospectively identified participants among adolescent and adult patients seeking healthcare at the Medical Observation (MO) unit at YGH from 5 October 2015 through 4 October 2016. Adolescent and adult patients aged ≥12 years with an oral temperature of ≥38° C seen at the MO unit were eligible for enrolment. Febrile patients returning for the same illness episode were excluded from the study.

### Clinical data and sample collection

Using a standardized case report form, trained medical graduates collected demographic, clinical history, and physical examination data from consenting participants. We assessed the participant’s severity status at the time of enrollment by calculating quick sequential organ failure assessment (qSOFA) score as defined by Sepsis-3 Task Force [15]. Accordingly, we assigned one point each for three clinical criteria: respiratory rate ≥22 per minute, systolic blood pressure ≤100 mmHg, and Glasgow coma scale <15. A high qSOFA was defined as a score ≥2 [15]. Provisional diagnoses of the hospital clinical team were recorded and coded using the International Statistical Classification of Diseases and Related Health Problems, 10th Revision (ICD-10) codes [16].

Prior to commencing hospital antimicrobial therapy and after skin cleansing and disinfection, team members collected blood from each consenting participant and 8–10 mL were inoculated aseptically into BacT/ALERT standard aerobic (SA) blood culture bottles (bioMérieux, Marcy l’Etoile, France).

### Blood culture, isolate identification, and antimicrobial susceptibility testing

We performed blood culture processing, isolate identification, and antimicrobial susceptibility testing (AST) at the Microbiology Laboratory Section, YGH. Blood culture bottles were assessed for volume adequacy by comparing the weight before and after inoculation. Inoculated BacT/ALERT SA bottles were loaded into the BacT/ALERT 3D 60 Microbial Detection system (bioMérieux, Marcy l’Etoile, France) and incubated at 37°C for 5 days. Bottles flagging positive were sub-cultured following standard methods. Positive bottles with no growth on subculture were tested for the presence of pneumococcal antigen using BinaxNOW *Streptococcus pneumoniae* antigen card rapid immunochromatographic assay (Alere Ltd., Auckland, New Zealand) [17]. Isolate identification and AST were done by VITEK2 Compact 60 system (bioMérieux, Marcy l’Etoile, France). Clinical and Laboratory Standards Institute (CLSI) criteria [18, 19] were used for AST interpretation at YGH and results were reported to YGH clinicians responsible for patient care. We tested all isolates that were identified by the VITEK2 as *Burkholderia* spp. by latex agglutination assay for *Burkholderia pseudomallei*, as previously described [20]. We classified blood culture isolates of commensal organisms and gram-negative bacteria that are unlikely to cause BSI as likely contaminants. Likely contaminants included *Achromobacter* spp., *Bacillus* spp. other than *B*. *anthracis*, *Brevibacterium* spp., coagulase-negative staphylococci, *Corynebacterium* spp. other than *C*. *diphtheriae*, *Dermacoccus* spp., *Kocuria* spp., and *Micrococcus* spp. [21]. AST for fungal isolates was not performed.

For external quality control, isolate identification and AST were confirmed at Southern Community Laboratories (SCL), Dunedin, New Zealand. Matrix assisted laser desorption ionization time of flight (MALDI-TOF) mass spectrometry (Microflex LT, Bruker Daltonics, Billerica, MA, USA) and the BD Phoenix system (Becton and Dickenson, Franklin Lakes, New Jersey, USA) were used. *B*. *pseudomallei* isolates was not confirmed at SCL since the organism could not be imported into New Zealand. The European Committee for Antimicrobial Susceptibility Testing (EUCAST) disc diffusion method was used for AST determination when the Phoenix system was unable to provide a result. E-tests (Thermo Fisher Scientific, Auckland, New Zealand) were used to determine the minimum inhibitory concentrations (MICs) of ciprofloxacin, nalidixic acid, and azithromycin for isolates identified as *S*. *enterica*. At SCL, EUCAST clinical breakpoints [22] were used for AST interpretation. Final AST results were interpreted according to EUCAST criteria where available [22]. Susceptibility results for antimicrobial agents against *Burkholderia* spp., and those for nalidixic acid and azithromycin for *S*. *enterica*, for which EUCAST breakpoints do not exist, were interpreted by CLSI criteria [19].

At SCL, screening for ESBL, AmpC, and carbapenemase production among gram-negative isolates and phenotypic confirmation were performed following EUCAST methods [23]. ESBL-, carbapenemase-, and AmpC-producing organisms were defined on the results of the phenotypic confirmatory tests [23]. For all bacteria except *S*. *enterica*, we defined multidrug resistance (MDR) as acquired non-susceptibility (resistant or intermediate susceptibility) to at least one agent in ≥3 antimicrobial classes and extensive drug resistance (XDR) as non-susceptibility to at least one agent in all but 2 or fewer antimicrobial classes [24]. The definition of MDR for *S*. *enterica* followed the conventional definition of resistance to ampicillin, chloramphenicol, and trimethoprim-sulfamethoxazole [25].

### Whole-genome sequencing and detection of antimicrobial resistance mechanisms, sequence types, and phylogenetic analysis

We performed whole-genome sequencing (WGS) for *Enterobacteriaceae* and *Streptococcus agalactiae*. DNA was extracted using the Nucleospin Microbial DNA kit (Machery Nagel, Düren, Germany) according to the manufacturer’s instructions [26]. Quality of DNA was assessed with the Nanodrop One spectrophotometer (Thermo Fisher Scientific, Auckland, New Zealand) and Qubit fluorometry (Thermo Fisher Scientific, Auckland, New Zealand). WGS of extracted DNA was performed by Illumina HiSeq (Illumina Inc., Melbourne, Australia) with 2x125bp PE v4 sequencing chemistry at New Zealand Genomics Ltd, Palmerston North, New Zealand. Bioinformatic analysis of WGS data was done with the Nullarbor pipeline version 1.2 [27] to detect antimicrobial resistance genes and to determine sequence types (STs) of isolates. Reference genomes used are listed in the S1 Table. To detect mutations in the quinolone resistance determining regions (QRDR) among ciprofloxacin-resistant *E*. *coli*, sequences of *gyrA*, *gyrB*, *parC*, and *parE* of isolates were compared to those of *E*. *coli* strain K-12 substrain MG1655 (GenBank accession number: NC_000913.3) using CLC sequence viewer version 8.0 (Qiagen, Hilden, Germany).

Phylogenetic analysis was performed as previously described [28]. Recombinant regions were filtered from the core genome SNP alignment and a maximum likelihood phylogenetic tree generated using Gubbins [29]. Trees were then rooted using the minimal ancestor deviation method [30].

We have submitted sequence data for *S*. *enterica* Typhi and Paratyphi A isolates to GenBank under BioProject PRJNA493305 [28] and *E*. *coli*, *K*. *pneumoniae*, and *S*. *agalactiae* to BioProject PRJNA624724. Accession numbers and metadata for *E*. *coli*, *K*. *pneumoniae*, and *S*. *agalactiae* are provided in supporting information (S1–S3 Appendices).

### Statistical methods

Clinical and laboratory data were entered into Microsoft Access 2013 and Excel 2013 (Microsoft Corporation, Redmond, Washington, USA). Data were analyzed using STATA 13.1 version (Stata Corporation, College Station, TX, USA). Descriptive data were calculated for continuous variables. Proportions of BSIs and antimicrobial resistance patterns of identified pathogens were calculated. The *X*^2^ test or Fisher’s exact test, as appropriate, were used to compare proportions.

We defined a BSI for any positive blood culture yielding pathogenic bacteria or fungi obtained from a febrile participant. For the purpose of statistical analyses, we classified BSIs as community onset BSI (CO BSI) and hospital-acquired BSI (HA BSI). CO BSI was defined as any BSI from outpatients or inpatients who came directly from the community to YGH, or had received <48 hours of healthcare at another healthcare facility, whereas HA BSI were defined as any BSI acquired at the transferring hospital where the participant was hospitalized for >48 hours [31]. To distinguish BSI originated in the community but with healthcare exposure other than hospital admission, we further subclassified CO BSI into healthcare-associated BSI (HCA BSI) and community-acquired BSI (CA BSI), defining each based on modifications of published definitions [32]. To calculate blood culture volume adequacy, we defined the manufacturer’s recommended volume of 10mL±20% or 2mL as ‘adequate.’ Blood volumes less than ‘adequate’ were regarded as ‘underfilled’ and those more than adequate as ‘over-filled.’ To assess seasonal variations of BSIs, we defined ‘wet season’ as May through October and ‘dry season’ as November through April [33].

We examined associations between participant qSOFA score on presentation at the MO unit and vital status at discharge. Odds ratios were calculated by univariate logistic regression to investigate associations between exposure and outcome variables, and to examine associations between season and proportions of pathogens isolated. The two-sided P value of <0.05 was considered significant.

## Results

### Study population, participant demographics, and clinical data

**Fig 2:**
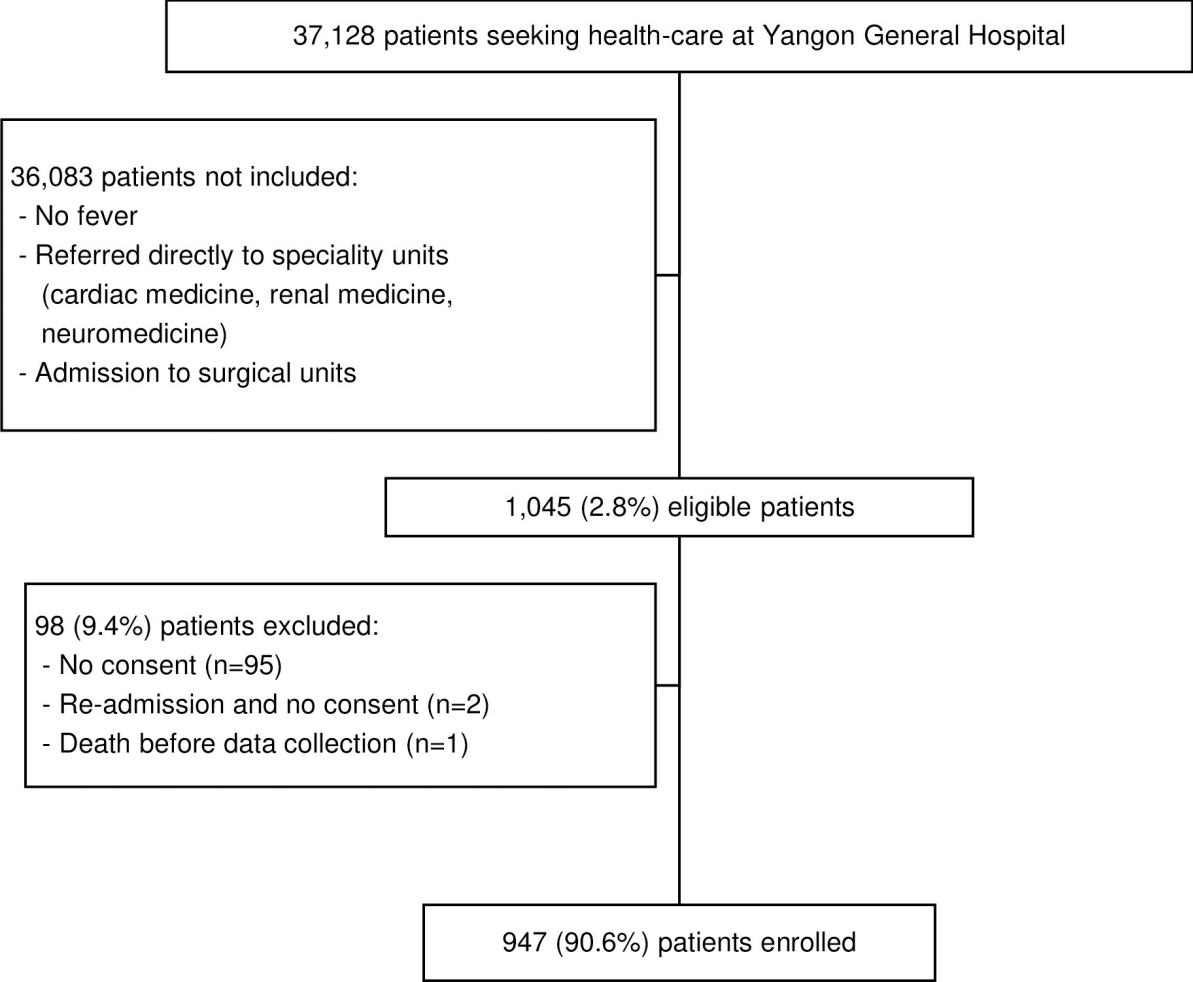
Flow diagram for enrolling febrile patients at Yangon General Hospital, 2015–2016.

Of 37,128 patients seen at the MO unit, YGH from 5 October 2015 through 4 October 2016, 1,045 (2.8%) were eligible for inclusion, among which 947 (90.6%) consented and were enrolled in the study (Fig 2). Of 947 participants, 671 (70.9%) resided in the Yangon Region, and 428 (45.2%) were transferred from other healthcare facilities. Among 428 participants transferred, 219 (51.2%) came from healthcare centers with inpatient facilities and 209 (50.8%) from rural health centers or private clinics. Participants’ demographics and clinical characteristics are shown in Table 1. The median (range) age of participants was 37 (12, 94) years and 446 (47.1%) were female. Of participants, comorbidities included 122 (12.9%) hypertension, 92 (9.7%) tuberculosis, and 80 (8.4%) diabetes mellitus (Table 1). ICD-10 admission diagnoses of study participants included 329 (34.7%) unspecified fever, 100 (10.6%) enteric fever, 62 (6.5%) lower respiratory tract infection, 49 (5.2%) meningitis or encephalitis, and 35 (3.7%) septicemia (Table 1).

**Table 1:** Demographics and clinical characteristics of febrile patients attending Yangon General Hospital, 2015–16.

Characteristic	Participants without BSI (n = 857)		Participants with BSI (n = 90)		Total participants (n = 947)	
	Median, (range)	n (%)	Median (range)	n (%)	Median (range)	n (%)
Age, years	38 (12, 87)		33 (13, 94)		37 (12, 94)	
Gender						
- Male		448 (52.3)		53 (58.9)		501 (52.9)
- Female		409 (47.7)		37 (41.1)		446 (47.1)
Duration of fever, days	5 (1, 30)		5 (1, 28)		5 (1, 30)	
Presenting symptoms						
- Fever (>38°C)		857 (100.0)		90 (100.0)		947 (100.0)
- Headache		357 (41.7)		47 (52.2)		404 (42.7)
- Cough		257 (30.0)		23 (25.6)		280 (29.6)
- Vomiting		240 (28.0)		35 (38.9)		275 (29.0)
- Diarrhoea		110 (12.8)		10 (11.1)		120 (12.7)
- Dysuria		79 (9.2)		9 (10.0)		88 (9.3)
Physical signs						
- Abdominal tenderness		86 (10.0)		16 (17.8)		102 (10.8)
- Jaundice		84 (9.8)		7 (7.8)		91 (9.6)
- Basal lung crepitations		66 (7.7)		7 (7.8)		73 (7.7)
- Hepatomegaly		46 (5.4)		7 (7.8)		53 (5.6)
- Ascites		30 (3.5)		2 (2.2)		32 (3.4)
- Neck stiffness		20 (2.3)		2 (2.2)		22 (2.3)
- Cardiac murmur		15 (1.8)		4 (4.4)		19 (2.0)
Severity assessment						
- Respiratory rate ≥22 rate per minute		178 (20.8)		25 (27.8)		203 (21.4)
- Glasgow coma scale <15		122 (14.2)		15 (16.7)		137 (14.5)
- Systolic blood pressure ≤100 mmHg		100 (11.7)		14 (15.6)		114 (12.0)
- qSOFA score ≥2		55 (6.4)		9 (10.0)		64 (6.8)
Comorbid conditions						
- Hypertension		112 (13.1)		10 (11.1)		122 (12.9)
- Tuberculosis		91 (10.6)		1 (1.1)		92 (9.7)
- Diabetes mellitus		72 (8.4)		8 (8.9)		80 (8.4)
- Cardiovascular diseases^*^		31 (3.6)		3 (3.3)		34 (3.6)
- Chronic liver disease^†^		15 (1.8)		4 (4.4)		19 (2.0)
- Malignancy		17 (2.0)		1 (1.1)		18 (1.9)
- Hematological malignancy		10 (1.2)		1 (1.1)		11 (1.2)
- Solid organ tumors		7 (0.8)		0 (0.0)		7 (0.7)
- HIV		14 (1.6)		0 (0.0)		14 (1.5)
- Renal disease		10 (1.2)		1 (1.1)		11 (1.2)
- Respiratory diseases^‡^		6 (0.7)		3 (3.3)		6 (0.6)
- Neurological diseases		5 (0.6)		1 (1.1)		9 (1.0)
Recent exposure to antimicrobial agents						
- No		591 (69.0)		29 (32.2)		620 (65.5)
- Yes		148 (17.3)		22 (24.4)		170 (17.9)
- Did not know		118 (13.8)		39 (43.3)		157 (16.6)
Admission diagnosis						
- Unspecified fever		293 (34.2)		36 (40.0)		329 (34.7)
- Enteric fever		88 (10.3)		12 (13.3)		100 (10.6)
- Lower respiratory tract infection		58 (6.8)		4 (4.4)		62 (6.5)
- Meningitis or encephalitis		47 (5.5)		2 (2.2)		49 (5.2)
- Septicemia		30 (3.5)		5 (5.6)		35 (3.7)
- Cerebrovascular disease		34 (4.0)		0 (0.0)		34 (3.6)
- Urinary tract infection		26 (3.0)		6 (6.7)		32 (3.4)
- Pulmonary tuberculosis		29 (3.4)		1 (1.1)		30 (3.2)
- Cirrhosis of liver		27 (3.2)		1 (1.1)		28 (3.0)
- Acute viral infection		24 (2.8)		3 (3.3)		27 (2.9)
- Dengue fever/Dengue hemorrhagic fever		18 (2.1)		2 (2.2)		20 (2.1)
- Acute gastroenteritis		14 (1.6)		3 (3.3)		17 (1.8)
- Infective endocarditis		12 (1.4)		4 (4.4)		16 (1.7)
- HIV infection		13 (1.5)		1 (1.1)		14 (1.5)
- Acute viral hepatitis A, B or C		5 (0.6)		6 (6.6)		11 (1.2)
- Leptospirosis		4 (0.5)		1 (1.1)		5 (0.5)
- Malaria		4 (0.5)		1 (1.1)		5 (0.5)
- Leprosy		3 (0.4)		0 (0.0)		3 (0.3)
YGH admission status									
- Admitted		763 (89.0)		87 (96.0)		850 (89.8)
- Treated as outpatient		94 (11.0)		3 (3.3)		97 (10.2)

BSI, Bloodstream infection; qSOFA, quick sequential (sepsis-related) organ failure assessment; HIV, human immunodeficiency virus; YGH, Yangon General Hospital.

^*^Cardiovascular diseases include heart failure, ischemic heart disease and valvular heart disease;

^†^Chronic liver diseases include alcoholic hepatitis, chronic viral hepatitis B or C, and cirrhosis of liver;

^‡^Respiratory diseases include asthma, chronic obstructive pulmonary disease, and pneumonia.

### Bloodstream infections, assessment of severity, and the vital status

Of 947 participants, 90 (9.5%) had BSI. The median (range) age of participants with BSI was 33 (13, 94) years and 37 (41.1%) were female (Table 1). Of 90 participants with BSI, 82 (91.1%) had CO BSI including 76 (84.4%) CA BSI and 6 (6.7%) HCA BSI. Eight (8.9%) had HA BSI.

Of all participants, 64 (6.8%) had a qSOFA score ≥2 (Table 1). Bloodstream infection was detected in 9 (14.0%) of 64 participants with qSOFA score ≥2 compared to 81 (9.2%) of 883 with score <2 (OR 1.62; 95% CI 0.77–3.40; p = 0.202). Vital status at discharge was assessed in 470 (49.6%) participants of which 64 (13.6%) died. Of 44 participants with known vital status and qSOFA score ≥2, 21 (47.7%) died compared to 45 (10.6%) of 426 with known vital status and qSOFA score <2 (OR 7.73; 95% CI 3.97–15.07; p<0.001). Thirteen (24.1%) of 54 participants with known vital status and BSI died compared to 53 (12.7%) of 416 participants with known vital status and who did not have BSI (OR 2.17; 95% CI 1.09–4.32; p = 0.027). BSI was found in 12 (12.0%) of 100 participants clinically diagnosed with enteric fever and 5 (16.7%) of 30 participants diagnosed to have septicemia on admission.

### Blood culture

#### Blood culture volume adequacy and pathogen isolation

Of 947 blood cultures, 90 (9.5%) yielded pathogens, 44 (4.6%) grew likely contaminants, and 15 (1.6%) flagged positive but did not grow on subculture. Among 888 bottles that neither grew contaminants nor showed growth on subculture, 84 (10.5%) of 799 adequately filled blood culture bottles grew pathogens compared to 6 (6.7%) of 89 underfilled bottles (OR = 1.63; 95% CI 0.69–4.69, p = 0.354). No bottles were over-filled.

#### Bloodstream isolates

From 90 positive blood cultures, 91 pathogens were isolated. On one occasion two pathogens, *Escherichia coli* and *Klebsiella pneumoniae*, were isolated from a single bottle. Among 91 pathogens isolated, 76 (83.5%) were gram-negative bacteria, 11 (12.1%) were gram-positive bacteria, and 4 (4.4%) were yeasts (Table 2). We confirmed 1 (33.3%) *B*. *pseudomallei* among 3 *Burkholderia* spp. by latex agglutination assay. Of 91 pathogens, 77 (84.6%) were isolated from CA BSI, 8 (8.8%) from HA BSI, and 6 (6.6%) from HCA BSI. All 43 (100%) *S*. *enterica* were isolated from participants with CA BSI.

**Table 2:** Bloodstream pathogenic isolates recovered from febrile patients admittd to Yangon General Hospital, 2015–2016.

Pathogens isolated	n (%)
**Gram-negative bacteria**	**76 (83.5)**
Enterobacteriaceae	70 (76.9)
*Salmonella enterica* spp.	43 (47.3)
*S*. *enterica* Typhi	33 (36.3)
*S*. *enterica* Paratyphi A	10 (11.0)
*Escherichia coli*	20 (22.0)
*Klebsiella pneumoniae*	7 (7.7)
Non-*Enterobacteriaceae*	6 (6.6)
*Acinetobacter* spp.	2 (2.2)
*Burkholderia cepacia* complex	2 (2.2)
*Burkholderia pseudomallei*	1 (1.1)
*Pseudomonas aeruginosa*	1 (1.1)
**Gram-positive bacteria**	**11 (12.1)**
*Staphylococcus aureus*	6 (6.6)
Beta-hemolytic Streptococcus^*^	2 (2.2)
*Enterococcus faecalis*	2 (2.2)
*Streptococcus anginosus*	1 (1.1)
**Yeast**	**4 (4.4)**
*Candida tropicalis*	2 (2.2)
*Candida albicans*	1 (1.1)
*Candida famata*	1 (1.1)
**Total**	**91 (100.0)**

^*^Beta-hemolytic streptococci included: *Streptococcus agalactiae* (n = 1) and *Streptococcus dysgalactiae* (n = 1).

#### Blood cultures with no growth on subculture

Among 15 blood culture bottles that flagged positive on the BacT/ALERT system but did not yield isolates on subculture, 1 (6.7%) tested positive for pneumococcal antigen. Gram-positive cocci in pairs were observed in the gram-stained smear of the pneumococcal antigen-positive blood-broth mixture whereas the remainder had negative gram-stains.

### Clinical data of participants with bloodstream infection

Among 100 participants with clinically suspected enteric fever, 11 (11.0%) had blood cultures positive for *S*. *enterica* Typhi or Paratyphi A. Thirty-two (74.4%) of 43 participants with blood culture-positive *S*. *enterica* were not identified as having enteric fever clinically. Among 43 participants with blood culture-confirmed *S*. *enterica* BSI, typhoid fever complications were observed in none. *S*. *agalactiae* was isolated from a 22 year-old previously healthy participant presenting with fever, headache, and neck stiffness.

### Seasonal pattern of bloodstream infections

Of 947 blood cultures, pathogens were isolated in 43 (10.2%) of 423 collected during the dry season whereas 47 (9.0%) were isolated from 524 in the wet season (OR 1.15; 95% CI 0.74–1.77; p = 0.533). *Enterobacteriaceae* were isolated from 34 (79.1%) of 43 positive blood cultures during the dry season and 35 (74.5%) of 47 positive blood cultures during the wet season (OR 1.29; 95% CI 0.848–3.47; p = 0.606). *S*. *enterica* was isolated from 24 (51.1%) of 47 positive blood cultures in the wet season whereas 19 (44.2%) were identified in 43 positive blood cultures during the dry season (OR 1.32; 95% CI 0.57–3.02; p = 0.514).

### Antimicrobial susceptibility of bloodstream isolates

The AST patterns of gram-negative pathogens isolated from blood cultures from febrile patients are shown in Table 3. Of 76 gram-negative bacteria, 22 (28.9%) were MDR and 2 (2.6%) were XDR (Table 4). All MDR and XDR bacteria were susceptible to colistin (Table 3). We have recently reported the AST pattern of *S*. *enterica* isolates [28]. Among gram-positive bloodstream isolates, no *S*. *aureus* were methicillin-resistant (MRSA). The *Enterococcus faecalis* and pathogenic streptococci were susceptible to all antimicrobial agents tested.

**Table 3:** Antimicrobial susceptibility pattern of gram-negative bloodstream isolates from febrile patients at Yangon General Hospital, 2015–2016.

	Pathogens, number (%) susceptible							
Antimicrobial agents	*S*. *enterica* Typhi*, n = 33	*S*. *enterica* Paratyphi A*, n = 10	*E*. *coli*, n = 20	*K*. *pneumoniae*, n = 7	*Acinetobacter* spp, n = 2	*B*. *cepacia* complex ^†^, n = 2	*B*. *pseudomallei* ^†^, n = 1	*P*. *aeruginosa*, n = 1
Ampicillin	33 (100)	10 (100)	1 (5)	0 (0)	NT	NT	1 (100)	NT
Amoxicillin-clavulanate	33 (100)	10 (100)	1 (5)	4 (57)	NT	NT	NT	NT
Piperacillin-tazobactam	33 (100)	10 (100)	14 (70)	5 (71)	0 (0)	2 (100)	1 (100)	1 (100)
Ceftriaxone	33 (100)	10 (100)	3 (15)	4 (57)	0 (0)^†^	NT	1 (100)	NT
Ceftazidime	33 (100)	10 (100)	4 (20)	4 (57)	0 (0) ^†^	2 (100)	1 (100)	1 (100)
Cefepime	33 (100)	10 (100)	3 (15)	5 (71)	2 (100)^†^	2 (100)	1 (100)	1 (100)
Aztreonam	33 (100)	10 (100)	3 (15)	4 (57)	NT	NT	NT	NT
Ertapenem	33 (100)	10 (100)	19 (95)	7 (100)	NT	NT	NT	NT
Imipenem	33 (100)	10 (100)	19 (95)	7 (100)	2 (100)	NT	NT	1 (100)
Meropenem	33 (100)	10 (100)	19 (95)	7 (100)	2 (100)	2 (100)	1 (100)	1 (100)
Amikacin	0 (0) ^‡^	0 (0) ^‡^	18 (90)	6 (86)	2 (100)	NT	NT	1 (100)
Gentamicin	0 (0) ^‡^	0 (0) ^‡^	10 (50)	5 (71)	2 (100)	NT	NT	0 (100)
Tobramycin	0 (0) ^‡^	0 (0) ^‡^	9 (45)	5 (71)	2 (100)	NT	NT	NT
Nalidixic acid	0 (0)	0 (0)	NT	NT	NT	NT	NT	NT
Ciprofloxacin	0 (0)	0 (0)	4 (20)	4 (57)	2 (100)	0 (0)	1 (100)	1 (100)
Azithromycin	33 (100)	10 (100)	NT	NT	NT	NT	NT	NT
Tigecycline	33 (100)	10 (100)	20 (100)	3 (43)	NT	NT	NT	NT
Tetracycline	33 (100)	10 (100)	4 (20)	4 (57)	1 (50) ^§^	NT	1 (100)	NT
Trimethoprim-Sulfamethoxazole	33 (100)	10 (100)	5 (25)	4 (57)	2 (100)	NT	1 (100)	NT
Chloramphenicol	33 (100)	10 (100)	11 (55)	5 (71)	NT	NT ^¶^	NT	NT
Colistin	33 (100)	10 (100)	20 (100)	7 (100)	2 (100)	NT	NT	NT

NT: Not tested.

*Results have recently been reported in a separate paper [28];

^†^AST interpretation according to CLSI guidelines [19];

^‡^Aminoglycosides (amikacin, gentamicin, tobramycin) are not effective clinically against *Salmonella* spp. and are recommended to be reported as resistant [19];

^§^Tested with doxycycline.

^¶^AST not tested since disk diffusion test is not reliable.

**Table 4:** Prevalence of phenotypic antimicrobial resistance patterns among gram-negative bloodstream pathogens isolated from febrile patients at Yangon General Hospital, 2015–2016.

Phenotypic resistance pattern	*E*. *coli*	*K*. *pneumoniae*	*Acinetobacter* spp	*S*. enterica*	Total
MDR	**(n = 20)**	**(n = 7)**	**(n = 2)**	**(n = 43)**	
XDR	**n (%)**	**n (%)**	**n (%)**	**n (%)**	**n**
MDR	17 (85)	3 (43)	2 (100)	0 (0)	22
XDR	2^†^(10)	0 (0)	0 (0)	0 (0)	2
ESBL	15 (75)	3 (43)	0 (0)	0 (0)	18

MDR, Multi-drug resistance; XDR, extensive drug resistance; ESBL, extended-spectrum beta-lactamase.

*Results reported in a separate paper [28];

^†^One ESBL-producer and one carbapenemase-producer.

### Phenotypic confirmation of extended-spectrum beta-lactamase, AmpC beta-lactamase, and carbapenemase production among *Enterobacteriaceae*

Of 70 *Enterobacteriaceae*, phenotypic testing identified ESBL-production in 18 (25.7%), AmpC beta-lactamase production in 1 (1.4%), and carbapenamase-production in 1 (1.4%) (Table 4). Of 17 MDR *E*. *coli*, ESBL-production was found in 14 (82.4%) and AmpC beta-lactamase in 1 (5.9%), while 2 XDR isolates produced an ESBL (n = 1) or a carbapenemase enzyme (n = 1). All 3 (100.0%) MDR *K*. *pneumoniae* produced an ESBL. No *S*. *enterica* produced an ESBL, AmpC, or carbapenemase.

Among non-*Salmonella Enterobacteriaceae*, ESBL-production was detected in 12 (63.2%) of 19 CA BSI, 2 (66.7%) of 3 HCA BSI, and 4 (80.0%) of 5 HA BSI. We identified an AmpC beta-lactamase-producing *E*. *coli* (n = 1) in a CA BSI. An *E*. *coli* isolate which produced a carbapenemase (n = 1) was recovered from a diabetic patient with HCA BSI who was previously admitted to a private healthcare center in Yangon for <48 hours.

### Antimicrobial resistance mechanisms among *Escherichia coli* and *Klebsiella pneumoniae* bloodstream isolates

We have recently reported the genomic analysis of the *S*. *enterica* isolates [28]. Here we report the genomic analysis of the *E*. *coli* and *K*. *pneumoniae* isolates. Of 27 *E*. *coli* and *K*. *pneumoniae* isolates, ESBL genes were identified in 19 (70.4%), including 18 with phenotypic evidence of ESBL production. An ESBL gene was also identified in the single carbapenemase-producing *E*. *coli* (Table 5). Of 19 *E*. *coli* and *K*. *pneumoniae* isolates that harbored an ESBL gene, *bla*_CTX-M 15_ (group 1 CTX-M) was detected in 17 (89.5%) while the remaining 2 (10.5%), both *E*. *coli*, harbored a group 9 CTX-M gene (1 *bla*_CTX-M 14_ and 1 *bla*_CTX-M 27_). We identified *bla*_CMY-42_ in the AmpC producer and *bla*_NDM-5_ in the carbapenemase-producing *E*. *coli*. (Table 5).

**Table 5:** Beta-lactamase genes identified in *Escherichia coli* and *Klebsiella pneumoniae* bloodstream isolates from febrile patients at Yangon General Hospital, 2015–2016.

Resistance genes	*E*. *coli*	*K*. *pneumoniae*	Total
	(n = 20)	(n = 7)	(n = 70)
	n (%)	n (%)	n (%)
ESBL			19 (27.1)
*bla*_CTX-M-15_	14^*^ (70.0)	3 (42.9)	17 (24.3)
*bla*_CTX-M-14_	1 (5.0)	0 (0.0)	1 (1.4)
*bla*_CTX-M-27_	1 (5.0)	0 (0.0)	1 (1.4)
Carbapenemase			
*bla*_NDM-5_	1 (5.0)	0 (0.0)	1 (1.4)
AmpC			
*bla*_CMY-42_	1 (5.0)	0 (0.0)	1 (1.4)

ESBL, extended spectrum beta lactamase.

^*^One isolate co-produced *bla*_NDM-5_

ESBL, extended spectrum beta lactamase. *An isolate co-produced *bla*_NDM-5._

Of 19 ciprofloxacin-resistant *E*. *coli* and *K*. *pneumoniae* isolates, mutations in QRDR were found in 16 (84.2%) while 14 (73.7%) had plasmid-mediated quinolone resistance (PMQR) genes. Among 16 isolates that possessed QRDR mutations, three types of mutations (two in *gyrA* and one in *parC*) were seen in 15 (93.8%) *E*. *coli* (S2 Table). Among *E*. *coli*, 11 (68.8%) of 16 ciprofloxacin-resistant isolates encoded PMQR genes, *aac-6’-Ib-cr* being the most prevalent (S2 Table). Only the PMQR gene *qnrB* was found in all 3 (100.0%) ciprofloxacin-resistant *K*. *pneumoniae* (S2 Table).

### Sequence type determination and phylogenetic analysis

Using WGS data, we confirmed that 6 (30.0%) of 20 *E*. *coli* were sequence type (ST) 131 and all harbored *bla*_CTX-M-15_. Phylogenetic analysis of the *E*. *coli* ST131 strains identified that our bloodstream isolates were not clonally related to each other (S1 Fig). Other *E*. *coli* sequence types included ST405 and ST648 and there were 3 (15.0%) isolates of each. Two each of *E*. *coli* ST405 and ST648 also carried *bla*_CTX-M-15._ One (50.0%) of 2 *E*. *coli* ST648 isolates harboring *bla*_CTXM-15_ also bore *bla*_NDM-5_. Among 7 *K*. *pneumoniae* isolates, we identified sequence types as 2 (28.6%) ST23, and 1 (14.3%) each of ST20, ST35, and ST307. We also identified that the single isolate of *S*. *agalactiae* belonged to the sequence type 283.

## Discussion

We found that *S*. *enterica* Typhi and Paratyphi A were collectively the most common cause of CA BSI at a tertiary referral hospital in Yangon, Myanmar. As previously reported, all *S*. *enterica* isolates were resistant to both nalidixic acid and ciprofloxacin, but were susceptible to all other antimicrobial classes tested [28]. ESBL-production was common among *E*. *coli* and *K*. *pneumoniae*. We also identified one isolate of carbapenemase-producing *E*. *coli* that was also XDR. One patient had a blood culture positive for *B*. *pseudomallei* and one for *S*. *agalactiae* ST283. Methicillin resistant *S*. *aureus* was not found in our study. *S*. *pneumoniae* antigen was detected in one of 15 positive blood culture bottles with no growth on subsequent subculture.

Among participants, 9.5% had blood cultures positive for pathogens. Previously, pathogen-positive blood cultures were detected in 28 (34.6%) of 81 patients who attended a military hospital in Yangon [34] and 111 (34.3%) of 324 hospitalized patients in Mandalay, situated in central Myanmar [35]. The lower prevalence of BSI in our study compared to previous studies is likely due to the inclusion of all febrile patients in this prospective study, independent of disease severity or clinical discretion.

The finding of *S*. *enterica* Typhi and Paratyphi A as the leading cause of BSI in Yangon is consistent with similar studies from other parts of South and South-East Asia [5, 36–38]. In a study of patients suspected to have enteric fever in Mandalay in central Myanmar during 2012–2013, *S*. *enterica* Typhi was isolated from 4.5% of blood cultures [35]. A study at Yangon Children’s Hospital (YCH) in 1998–1999 identified *S*. *enterica* Typhi as the most common cause of pediatric BSI, accounting for 43.1% bloodstream pathogens [6]. In high typhoid fever incidence settings, typhoid incidence tends to be highest among infants and young children [37, 39]. Therefore, the YCH finding is not surprising and consistent with other work suggesting that typhoid fever incidence is high in Yangon [14].

Fluoroquinolones have become the mainstay of enteric fever management in South and South-East Asia, including in Myanmar, in response to the emergence of MDR *S*. *enterica* Typhi [40]. MDR *S*. *enterica* Typhi was identified in Myanmar more than a decade ago [6], prompting a switch to the fluoroquinolone ciprofloxacin for the treatment of suspected enteric fever. We demonstrate that contemporary *S*. *enterica* isolates were resistant to nalidixic acid and ciprofloxacin, yet susceptible to ampicillin, chloramphenicol, and trimethoprim-sulphamethoxazole [28]. Notably, neither nalidixic acid nor ciprofloxacin resistance was detected in *S*. *enterica* isolates from the YCH study in 1998 [6]. However, by 2012 a quarter of *S*. *enterica* bloodstream isolates from Mandalay were nalidixic acid resistant [35] and by 2014 half of *S*. *enterica* isolates at YGH were ciprofloxacin-resistant [41]. Nalidixic acid susceptibility testing was not done in a 2014 YGH study [42]. As supported by our recent genomic analysis [28], these changes are likely due to the recent emergence and spread of fluoroquinolone-resistant typhoidal *S*. *enterica* in Myanmar, and possibly due to lack of ascertainment of unrecognized nalidixic acid-resistance and changes in fluoroquinolone susceptibility interpretive criteria [43].

Azithromycin is an alternative option for the treatment of uncomplicated MDR and fluoroquinolone-resistant *S*. *enterica* Typhi infections [44]. Reports of azithromycin treatment failure have been noted between 2004 and 2005 in Vietnam [45] and during 2016 in India [46] associated with azithromycin MICs 2–8 μg/mL. The range of azithromycin MIC for *S*. *enterica* isolates in our study was 2–12 μg/mL. YGH treatment guidelines recommend the use of azithromycin or an extended-spectrum cephalosporin for the management of enterica fever due to ciprofloxacin-resistant *S*. *enterica*. Since we could not access the antimicrobial treatment given at YGH for all participants, we could not correlate treatment outcome among study participants with azithromycin MIC. Likewise, ESBL-producing, ceftriaxone-resistant *S*. *enterica* Typhi is the cause of an ongoing outbreak of typhoid fever in Pakistan that began in 2016 [9]. Although ESBL production has not been reported in *S*. *enterica* Typhi isolates from Myanmar, its emergence might be curtailed by judicious use of antimicrobials in the community and its spread by early identification and intervention with vaccine.

We identified CO BSI due to ESBL-producing *Enterobacteriaceae*, as well as MDR and XDR phenotypes. Unsurprisingly, the proportion of isolates producing an ESBL and with a XDR phenotype was smaller than our previous retrospective study that included HA BSI [41] and lower than other studies including HA BSI from YGH performed in early 2015 [47] and from North Okkalapa Hospital (NOGH), Yangon, in 2016 [48]. Nonetheless, the availability of antimicrobial agents without prescription over the counter [49] and use of antimicrobial agents in food animals [50] may contribute to the development of AMR among pathogens acquired in the community.

We identified ESBL genes in nearly three quarters of *E*. *coli* and *K*. *pneumoniae* bloodstream isolates, most commonly *bla*_CTX-M-15_. Previously, we reported *bla*_CTX-M-15_ in just over 70% of ESBL-producing *Enterobacteriaceae* isolated from adult inpatients with BSI at YGH in 2014 [41]. Our findings are consistent with the global dissemination of *bla*_CTX-M-15_ among gram-negative bacteria [51]. This gene is frequently detected among *E*. *coli* and *K*. *pneumoniae* isolated from other Asian countries [12, 52]. In addition, *E*. *coli* ST131 commonly encodes *bla*_CTX-M-15_ and has been associated with community-onset infections [51]. The detection of the globally disseminated *bla*_CTX-M-15_ gene among ESBL-producers, including the pandemic *E*. *coli* ST131, in our study warrants further investigations in other areas of Myanmar.

We identified *bla*_NDM-5_ in a single *E*. *coli* isolate that phenotypically produced a carbapenemase. Notably, *bla*_NDM-5_ was not detected in our previous retrospective study at YGH in 2014 [41]. Our finding in the present study is consistent with those from two Myanmar studies done at YGH in early 2015 [53] and at NOGH in Yangon in 2016 [48] in which *bla*_NDM-5_ was detected among the majority of *E*. *coli* investigated. Isolates bearing *bla*_NDM-5_ have been detected increasingly in Asian countries [54–57] and among travelers to the Indian subcontinent [58, 59]. Identification of *bla*_NDM-5_ from an HCA BSI associated with a private hospital, in our prospective study raises concerns that *bla*_NDM-5_ *E*. *coli* strains may be widespread both in public and private hospitals in Myanmar. Of concern, NDM-5 producing *E*. *coli* also bore *bla*_CTX-M-15_ and other antimicrobial resistance genes. This suggests possible transfer of plasmids with multiple resistance genes or presence of multiple plasmids carrying AMR genes.

The pandemic sequence type *E*. *coli* ST131 was predominant among community-onset *E*. *coli* BSI in our study. *E*. *coli* ST131 has been reported in several Asian and South-East Asian countries [52]. Such strains often express *bla*_CTX-M-15_ [51, 52], are more likely to be multidrug resistant than other ESBL-producing *E*. *coli* [51], and are more likely to be associated with community-acquired infections [51]. Although less common, carriage of *bla*_CTX-M-15_ has also been identified in *E*. *coli* ST38, ST405, and ST648 [51]. Similarly, we identified that all six *E*. *coli* ST131 acquired in the community carried the *bla*_CTX-M-15_ gene and were MDR, and *bla*_CTX-M-15_ was also carried by *E*. *coli* with STs 405 and 648. Consistent with other research [54, 58, 59], one of our *E*. *coli* ST648 isolates that carried *bla*_CTXM-15_ also bore *bla*_NDM-5_. Notably, *E*. *coli* ST648 is an emerging lineage associated with MDR [60]. This suggests that newly emerged *E*. *coli* associated with MDR is a cause of BSI in Myanmar.

Mutations in the QRDR, most commonly in the form of the Ser83Phe mutation, was often associated with fluoroquinolone resistance among *Enterobacteriaceae* in our study. This mutation was common in *E*. *coli* isolates, and as we have previously reported, was found in all *S*. *enterica* [28]. Some ciprofloxacin-resistant *E*. *coli* harbored *aac-6’-Ib-cr*, the most prevalent PMQR, in addition to QRDR mutations. The contemporary Myanmar study performed at the NOGH also detected *aac-6’-Ib-cr* as the major PMQR found in ciprofloxacin-resistant *E*. *coli* [48]. The NOGH study did not investigate QRDR mutations [48]. The plasmid-mediated *aac-6’-Ib-cr* is a variant of *aac-6’-Ib* that encodes aminoglycoside acetyltransferase enzyme and possess the ability to reduce the activities of both aminoglycosides and fluoroquinolones [61]. The gene has been widely identified among ciprofloxacin-resistant *E*. *coli* [61–63]. Our findings point to a need to avoid extensive use of fluoroquinolones to prevent further spread of resistance in the community and to consider alternative drug regimens.

Few gram-positive pathogens were isolated in our study. No MRSA was found. All *Streptococcus* spp. and *E*. *faecalis* were susceptible to all antimicrobial agents tested. *S*. *pneumoniae* were not isolated from blood cultures, although one positive blood culture that failed to grow on subculture was positive by the Binax antigen test. This might suggest that *S*. *pneumoniae* is an uncommon cause of BSI in Yangon. No published studies on invasive pneumococcal disease in Myanmar are available. Based on evidence that pneumococcal disease is common in children <5 years of age [64], pneumococcal conjugate vaccine was first introduced into the Myanmar national expanded programme of immunization in 2016. Our inability to immediately subculture blood culture bottles that flagged positive overnight or on weekends may have hindered our ability to isolate *S*. *pneumoniae* because of autolysis [65]. This is a common problem in laboratories with limited staff and resources [17]. Furthermore, 17.9% of participants reported antimicrobial use prior to enrollment, potentially lowering blood culture sensitivity. One blood culture, which signaled positive by the BacT/ALERT and in which gram-positive cocci were observed by microscopy, was positive for *S*. *pneumoniae* cell wall antigen by the Binax antigen test. This test has been suggested as an alternative means of detecting *S*. *pneumoniae* when there is no growth on subculture [17]. We recognize that the use of blood culture alone for the diagnosis of pneumococcal diseases underestimates its prevalence. As such, we recommend the future use of antigen detection as a bed-side test on urine samples when pneumococcal disease is clinically suspected or as an adjunct for hemolyzed blood cultures in clinical laboratories.

We isolated and identified two beta-hemolytic streptococci, including one isolate of *S*. *agalactiae* or Lancefield group B streptococcus (GBS). GBS is the leading cause of neonatal sepsis and meningitis in babies born to mothers with genital tract colonization [66], and is associated with puerperal sepsis in pregnant mothers [67]. It also causes invasive diseases in the elderly [68] and the immunocompromised [69]. Specific serotypes of *S*. *agalactiae* have been identified to cause serious infections in non-pregnant and otherwise healthy adults in Asia [70–72]. *S*. *agalactiae* ST283 was found to be associated with severe infections after consumption of raw freshwater fish in Singapore [71] and Lao PDR [72]. The GBS isolate from our study belonged to ST283. To our knowledge, detection of *S*. *agalactiae* ST283 has not been reported from either humans or aquatic animals in Myanmar (Kay Lwin Tun, pers comm, April 2019). Consumption of raw fish is uncommon in Myanmar [73, 74]. However, farmed fish is a growing source of protein in Asia [75] and the consumption of raw fish is increasingly popular in some areas [76, 77] including Myanmar [78]. The isolation of *S*. *agalactiae* ST283 in our study warrants greater attention to this pathogen in both people and fish in Myanmar.

Our study had a number of limitations. First, study participants seeking healthcare at a tertiary hospital in a large city were enrolled. Therefore, findings may not represent BSI among patients admitted to district level hospitals in areas other than Yangon. Second, since participants transferred from hospitals other than YGH were included in the study not all BSIs met the definition of community-acquired or community-onset. Third, nearly 20% of participants had taken antimicrobial agents before seeking healthcare at YGH, potentially reducing blood culture sensitivity. Fourth, our study did not include pediatric patients. Similar research is warranted among patients <12 years of age. Fifth, performing single aerobic blood culture lacks sensitivity for detection of bacteremia and will miss anaerobic bacteremia [79, 80]. Sixth, the lack of longitudinal clinical data and additional blood cultures meant that we may have classified some organisms as contaminants that were causing bacteremia. Finally, participants did not systematically undergo HIV testing or a test for blood parasites, so we were unable to examine potentially important associations between these co-infections and bacteremia.

In summary, we found that typhoidal *S*. *enterica* was the most common cause of CA BSI among febrile patients attending a large tertiary referral hospital in Yangon, Myanmar. The absence of MDR typhoidal *Salmonella* and the high prevalence of fluoroquinolone resistance is notable. This finding has important implications for the empiric management of enteric fever in Yangon and underscores the value of CA BSI surveillance to monitor the pattern of infecting organisms, serovars, and antimicrobial susceptibility. The emergence of ESBL- and carbapenemase-producing gram-negative bacteria carrying globally prevalent drug resistance genes among CO BSI highlights the need not only to redouble efforts to control antimicrobial-resistant organisms in healthcare facilities, but also to prevent their emergence and spread in the community. Isolation of ESBL- and NDM-producing *Enterobacteriaceae* from CO BSI also highlights the need to better understand the acquisition, carriage, and mode of transmission of antimicrobial resistant organisms outside the hospital setting. We recommend extending surveillance of AMR to include other common locations for healthcare seeking among all age groups in Myanmar, including primary healthcare centers and private hospitals. In so doing, we will gain a better understanding of antimicrobial resistance in the community setting which may help in the development of the national antimicrobial resistance plan.

## Supporting information

S1 Appendix Sequence quality, MLST, and metadata of *Escherichia coli* bloodstream isolates from febrile patients attending Yangon General Hospital, Yangon, Myanmar, 2015–2016. (XLSX)

S2 Appendix Sequence quality, MLST, and metadata of *Klebsiella pneumoniae* bloodstream isolates from febrile patients attending Yangon General Hospital, Yangon, Myanmar, 2015–2016. (XLSX)

S3 Appendix Sequence quality and MLST of *Streptococcus agalactiae* ST283 isolated from a febrile patient attending Yangon General Hospital, Yangon, Myanmar, 2015–2016. (XLSX)

S1 Table Reference genomes and plasmids used for bioinformatic analysis of whole-genome sequencing data from *Escherichia coli, Klebsiella pneumoniae*, and *Streptococcus agalactiae* causing bloodstream infections in febrile patients attending Yangon General Hospital, Yangon, Myanmar, 2015–16. (DOCX)

S2 Table Ciprofloxacin MIC and fluoroquinolone resistance mechanisms identified among *Escherichia coli* and *Klebsiella pneumoniae* bloodstream isolates from febrile patients attending Yangon General Hospital, Yangon, Myanmar, 2015–2016. (DOCX)

S3 Table Genes for resistance to antimicrobial agents other than beta-lactams and quinolones identified among *Escherichia coli* and *Klebsiella pneumoniae* bloodstream isolates from febrile patients attending Yangon General Hospital, Yangon, Myanmar, 2015–2016. (DOCX)

S1 Fig Core genome SNP phylogeny of *Escherichia coli* ST131 isolates from febrile patients attending Yangon General Hospital, Yangon, Myanmar. The presence or absence of mutations in the QRDR, PMQR genes, and the extended-spectrum beta-lactamase gene *bla*_CTXM-15_ are shown on the right. 3044 core genome SNPs were identified. A maximum likelihood tree was inferred from core genome SNPs, and rooted using the minimal ancestor deviation method [30]. The scale bars represent the phylogenetic distance of 10 SNPs. MIC, minimum inhibitory concentration; QRDR, quinolone resistance-determining region; PMQR, plasmid-mediated quinolone resistance; ESBL, extended-spectrum beta-lactamase; SNP, single nucleotide polymorphism.
(TIF)
