# Supplementary material for: A prospective study of bloodstream infections among febrile adolescents and adults attending Yangon General Hospital, Yangon, Myanmar
Source: PLoS Negl Trop Dis. 2020 Apr 30;14(4):e0008268. doi: 10.1371/journal.pntd.0008268 (PMC7217485; doi:10.1371/journal.pntd.0008268)
Supplement: S1 Table — (DOCX) [file pntd.0008268.s004.docx]

S1 Table. Reference genomes and plasmids used for bioinformatic analysis of whole-genome sequencing data from *Escherichia coli, Klebsiella pneumoniae* and *Streptococcus agalactiae* causing bloodstream infections in febrile patients attending Yangon General Hospital, Yangon, Myanmar, 2015-2016

| **Organism** | **Reference genome and plasmid** | **GenBank accession no.** |
| --- | --- | --- |
| *E. coli* | *E. coli* ST131 strain EC958, complete genome | NZ_HG941718.1 |
| *K. pneumoniae* | *K. pneumoniae* subsp. pneumoniae HS11286 chromosome | NC_016845/CP003200.1 |
|  | *K. pneumoniae* subsp. pneumoniae HS11286 plasmid pKPHS1 – pKPHS6 | NC_016838 – NC_016841; NC_016846, NC_016847 |
| *Streptococcus agalactiae* | *Streptococcus agalactiae* strain SGEHI2015-107 chromosome, complete genome | NZ_CP025027.1 |
