# Supplementary material for: A prospective study of bloodstream infections among febrile adolescents and adults attending Yangon General Hospital, Yangon, Myanmar
Source: PLoS Negl Trop Dis. 2020 Apr 30;14(4):e0008268. doi: 10.1371/journal.pntd.0008268 (PMC7217485; doi:10.1371/journal.pntd.0008268)
Supplement: S2 Table — (DOCX) [file pntd.0008268.s005.docx]

**S2 Table. Ciprofloxacin MIC and fluoroquinolone resistance mechanisms identified among *Escherichia coli* and *Klebsiella pneumoniae* bloodstream isolates from febrile patients attending Yangon General Hospital, Yangon, Myanmar during 2015-2016**

|  | **Ciprofloxacin MIC (mg/L)** | **MIC interpretation^*^** | **Isolates** | | **Fluoroquinolone resistance mechanism** | | |
| --- | --- | --- | --- | --- | --- | --- | --- |
|  |  |  | **N** | **(%)** | **QRDR mutation** | | **PMQR genes** |
|  |  |  |  |  | ***gyrA*** | ***parC*** |  |
| *Escherichia coli* (n=20) | >2 | R | 7 | (35.0) | Ser83Phe, Asp87Asn | Ser80Ile | *aac-6'-Ib-cr* |
|  | >2 | R | 5 | (25.0) | Ser83Phe, Asp87Asn | Ser80Ile | ND |
|  | >2 | R | 1 | (5.0) | Ser83Phe, Asp87Asn | Ser80Ile | *qepA1* |
|  | >2 | R | 1 | (5.0) | Ser83Phe, Asp87Asn | Ser80Ile | *qepA2* |
|  | >2 | R | 1 | (5.0) | Ser83Phe, Asp87Asn | Ser80Ile | *aac-6'-Ib-cr*, *qepA2*, *qnrB6* |
|  | 1 | R | 1 | (5.0) | Asp87Asn | ND | *qnrS1* |
|  | 0.25 | S^†^ | 2 | (10.0) | ND | ND | *qnrS1* |
|  | 0.25 | S^†^ | 1 | (5.0) | Asp87Asn | ND | ND |
|  | 0.125 | S^†^ | 1 | (5.0) | ND | ND | ND |
| *Klebsiella pneumoniae* (n=7) | >1 | R | 3 | (42.9) | ND | ND | *qnrB* |
| ^*^EUCAST clinical breakpoints [1]; ^†^Epidemiologic cut-off (ECOFF) value = 0.125 mg/L. MIC, minimum inhibitory concentration; QRDR, quinolone resistance-determining region; *gyrA*, DNA gyrase subunit A; *parC*, DNA topoisomerase IV subunit A; Ser, serine; Phe, phenylalanine; Asp, aspartic acid; Asn, asparagine; Ile, isoleucine; R, resistant; S, susceptible; ND, not detected. | | | | | | | |

1. The European Committee on Antimicrobial Susceptibility Testing. Breakpoint tables for interpretation of MICs and zone diameters Version 7.1. 2017. [cited 2017]. Available from: <http://www.eucast.org/clinical_breakpoints>.
