# Supplementary material for: A prospective study of bloodstream infections among febrile adolescents and adults attending Yangon General Hospital, Yangon, Myanmar
Source: PLoS Negl Trop Dis. 2020 Apr 30;14(4):e0008268. doi: 10.1371/journal.pntd.0008268 (PMC7217485; doi:10.1371/journal.pntd.0008268)
Supplement: S3 Table — (DOCX) [file pntd.0008268.s006.docx]

**S3 Table. Genes for resistance to antimicrobial agents other than beta-lactams and quinolones identified among *Escherichia coli* and *Klebsiella pneumoniae* bloodstream isolates from febrile patients attending Yangon General Hospital, Yangon, Myanmar, 2015-2016**

| **Antimicrobial agent** | **Antimicrobial resistance genes** | ***Escherichia coli*, n=20** | | ***Klebsiella pneumoniae*, n=7** | |
| --- | --- | --- | --- | --- | --- |
|  |  | **n** | **(%)** | **n** | **(%)** |
| Aminoglycoside | *aac-3-IIa*  *aac-3-IId*  *aac-6'-Ib-cr* ^*^  *aadA1*  *aadA2*  *aadA5*  *aadA16*  *rmtB*  *rmtE*  *strA*  *strB* | 6  4  8  0  4  8  1  1  0  8  9 | (30.0)  (20.0)  (40.0)  (0.0)  (20.0)  (40.0)  (5.0)  (5.0)  (0.0)  (40.0)  (45.0) | 1  1  3  1  0  0  2  0  1  3  3 | (14.3)  (14.3)  (42.9)  (14.3)  (0.0)  (0.0)  (28.6)  (0.0)  (14.3)  (42.9)  (42.9) |
| Tetracycline | *tetA*  *tetB* | 10  6 | (50.0)  (30.0) | 3  0 | (42.9)  (0.0) |
| Chloramphenicol | *catA1*  *catA2* | 3  4 | (15.0)  (20.0) | 0  1 | (0.0)  (14.3) |
| Trimethoprim-sulfamethoxazole | *dfrA1*  *dfrA7*  *dfrA12*  *dfrA14*  *dfrA17*  *dfrA27* | 1  1  4  4  6  1 | (5.0)  (5.0)  (20.0)  (20.0)  (30.0)  ((5.0) | 0  0  1  1  0  2 | (0.0)  (0.0)  (14.3)  (14.3)  (0.0)  (28.6) |
|  | *sul1*  *sul2* | 12  8 | (60.0)  (40.0) | 2  2 | (28.6)  (28.6) |
| Azithromycin | *mphA* | 13 | (65.0) | 2 | (28.6) |
| Multidrug efflux pump | *oqxA*  *oqxB* | 0  0 | (0.0)  (0.0) | 7  7 | (100.0)  (100.0) |
| ^*^Also encodes resistance to ciprofloxacin. | | | | | |
