# Supplementary figures and images for: A prospective study of bloodstream infections among febrile adolescents and adults attending Yangon General Hospital, Yangon, Myanmar
Source: PLoS Negl Trop Dis. 2020 Apr 30;14(4):e0008268. doi: 10.1371/journal.pntd.0008268 (PMC7217485; doi:10.1371/journal.pntd.0008268)

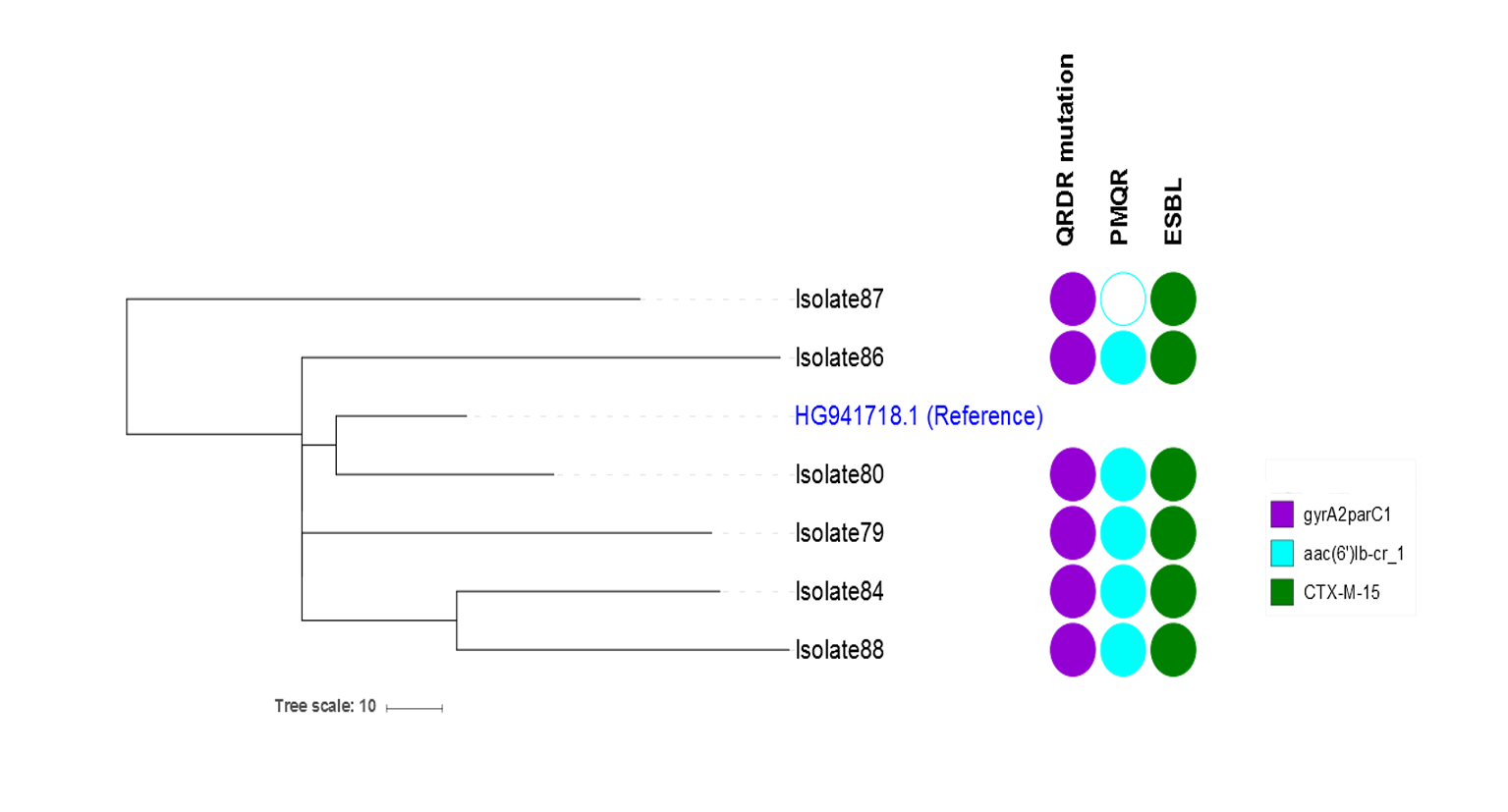

Supplement: S1 Fig — The presence or absence of mutations in the QRDR, PMQR genes, and the extended-spectrum beta-lactamase gene blaCTXM-15 are shown on the right. 3044 core genome SNPs were identified. A maximum likelihood tree was inferred from core genome SNPs, and rooted using the minimal ancestor deviation method [30]. The scale bars represent the phylogenetic distance of 10 SNPs. MIC, minimum inhibitory concentration; QRDR, quinolone resistance-determining region; PMQR, plasmid-mediated quinolone resistance; ESBL, extended-spectrum beta-lactamase; SNP, single nucleotide polymorphism. (TIF) [file pntd.0008268.s007.tif]
